# A Comparative Analysis of Liquid-Based Cytology and Conventional Smears in Fine-Needle Aspirates of Thyroid Lesions

**DOI:** 10.7759/cureus.45353

**Published:** 2023-09-16

**Authors:** Malti K Maurya, Rita Yadav, Madhu Kumar, Hitendra P Singh, Anand Mishra, Madhu Mati Goel

**Affiliations:** 1 Department of Pathology, King George’s Medical University, Lucknow, IND; 2 Department of Pathology, Prasad Institute of Medical Sciences, Lucknow, IND; 3 Department of Otolaryngology, Head and Neck Surgery, King George’s Medical University, Lucknow, IND; 4 Department of Endocrine Surgery, King George’s Medical University, Lucknow, IND; 5 Department of Pathology and Laboratory Medicine/Histopathology Cytopathology, Immunohistochemistry and Molecular Pathology, Medanta Hospital, Lucknow, IND

**Keywords:** liquid-based cytology, surepath, lymphocytic thyroiditis, colloid goiter, thyroid lesion, conventional smear cytology

## Abstract

Background

Palpable nodules in the thyroid are present in 4-7% of the general population. Fine-needle aspiration cytology is a safe and cost-effective method of choice for evaluating thyroid nodules. Aspirated samples can be manually spread directly onto the slide and stained in the conventional smear method. The liquid-cased cytology method has been recently introduced, which is an automated machine-based method, yielding a single slide with a clean background and greater preservation of cells and consuming less time for screening. This study aimed to compare the cytomorphological features and diagnostic accuracy of conventional smears and liquid-based cytology smears.

Methodology

This prospective study comprised 250 cases of thyroid lesions. Fine-needle aspiration cytology using conventional smears and liquid-based cytology smears was reported per the Bethesda system of reporting thyroid cytopathology. Detailed cytomorphological features were evaluated and compared in both techniques.

Results

The cellularity of conventional smears was significantly higher for scores 2+ and 3+ than paired liquid-based cytology smears (paired t-test, p < 0.001). The overall diagnostic efficacy of conventional smears and liquid-based cytology smears was equivalent in the majority of cases (n = 171, 68.4%). Conventional smears were better than liquid-cased cytology smears in 34 (13.6%) cases, and liquid-based cytology smears were better than conventional smears in eight (3.2%) cases. Liquid-based cytology smears showed a higher unsatisfactory rate compared to conventional Smears (15.6% vs. 5.2%). The sensitivity and specificity of conventional smears were 84.6% and 94.4%, respectively, compared to 68.7% and 92.4%, respectively, of liquid-based cytology smears.

Conclusions

Conventional smears are a cost-effective and easy method for diagnosing thyroid nodules. Liquid-based cytology smears can be used in association with conventional smears to enhance the accuracy of the evaluation of malignant thyroid nodules.

## Introduction

Palpable nodules in the thyroid are present in 4-7% of the general population, and 95% of these lesions are benign [1]. Fine needle aspiration (FNA) cytology using the conventional smear (CS) method is a diagnostic test of choice for the management of thyroid lesions. Although it is a simple, easy, and inexpensive method, it has some drawbacks such as bloody background, uneven spreading, entrapment of cells within the blood clot, and drying artifacts. The new liquid-based cytology (LBC) technique has almost replaced the conventional Papanicolaou (Pap) smear in the field of gynecologic cytology. People are now trying to use this technique for non-gynecological cytology samples, including the breast, salivary glands, and thyroid [1-4]. LBC is an automated machine-based technique that yields a single Pap-stained slide with a circular and evenly spread material in the center. It gives an advantage to monolayered cells with well-preserved morphology on a clean background and consumes less screening time. Few studies have been reported in the literature comparing the above two preparatory methods for the interpretation of thyroid lesions, with most of them reported from developed countries [2-6]. Hence, this study aimed to compare the efficacy of LBC smears versus CSs in the fine needle aspirate samples of thyroid lesions.

## Materials and methods

This prospective, observational study was conducted in the Department of Pathology at King George’s Medical University, Lucknow, India. The sample consisted of 250 cases of thyroid lesions. The study duration was one year, and it was approved by the Institutional Ethical Committee of King George’s Medical University (approval number: 3291/R Cell 13). All patients who came to our FNA outpatient department with palpable thyroid lesions were selected for the study, and informed consent was taken before the procedure. FNA was done using a 22-23-gauge needle attached to a 20 mL syringe in a Cameco syringe holder. A minimum of two to three passes were performed to obtain adequate material. Immediately after aspiration, the material was extruded onto the slides and one to three CSs were made and fixed in 95% ethanol and others were left to be air-dried. Material from the second pass was evacuated with needle rinse in a vial containing CytoRich™ red fixative solution for LBC preparation. In half of the cases, LBC was taken in the first pass while the other half was taken in the second pass to minimize sampling bias. CSs were stained with hematoxylin and eosin (H&E) stain, Pap stain, and May-Grunwald Giemsa stain. For the preparation of the LBC smear, the sample was processed using the Becton and Dickson SurePath™ system. After processing, a single pap-stained smear with homogenized material in the center of the slide was obtained. Both CS and LBC smears were evaluated by two pathologists independently. For each case, two to three slides of CSs and a single smear of LBC were screened for adequacy criteria according to the Bethesda System of Reporting Thyroid Cytopathology (TBSRTC) [6]. Adequacy criteria were at minimum six groups of benign, well-visualized, follicular cells. Each group was composed of at least 10 cells on a single slide. Representative smears of both preparations (CS and LBC) were diagnosed and compared based on cytological criteria, including cellularity, informative background (colloid, cyst macrophages), cell architecture, nuclear and cytoplasmic details, background blood, and ease of diagnosis. These scoring criteria were taken from previous studies except for one criterion, i.e., ease of interpretation. The ease of interpretation is the authors’ own parameter. The comparison was based on a semi-quantitative scoring system, as shown in Table 1.

**Table 1:** Cytological features and their scoring criteria CS: conventional smear; LBC: liquid-based cytology

Cytological features	Scores			
	0	1	2	3
Cellularity	Absent	Scanty	Adequate	Abundant
Cell architecture	Non-recognized	Moderately recognized	Well-recognized	N/A
Nuclear details	Poor	Fair	Good	Excellent
Cytoplasmic details	Poor	Fair	Good	Excellent
Informative background (colloid and cyst macrophages)	Absent	Present	N/A	N/A
Background blood	Absent	Mild	Moderate	Abundant
Ease of interpretation	N/A	CS = LBC	CS > LBC	LBC > CS

Data were analyzed using PRISM GraphPad software. The chi-square test, Fisher exact test, and kappa measure of the agreement were used to compare the data. The diagnostic efficacy of the two tests was assessed in terms of sensitivity, specificity, positive predictive value (PPV), negative predictive value (NPV), and diagnostic accuracy.

## Results

In this study, a total of 250 cases of thyroid lesions were evaluated by FNA cytology using CS and their corresponding LBC smears. There were 224 (89.6%) females and 26 (10.4%) males with an age range of 12-72 years. All cases were reported according to TBSRTC. The unsatisfactory rate was 5.2% (13 cases) among CS preparations. A maximum number of cases (81.2%) were classified as benign under category II, including colloid goiter, colloid cyst, colloid goiter with hyperplastic nodule, and lymphocytic thyroiditis. There were four (1.6%) cases in category III and 14 (5.6%) cases in category IV, including 13 follicular neoplasms and one Hurthle cell neoplasm. Thirteen (5.2%) cases were categorized in category VI, i.e., malignant thyroid lesions. In this group, papillary carcinoma (eight) was the most common, followed by anaplastic carcinoma (four) and medullary carcinoma (one), as shown in Table 2.

**Table 2:** Distribution of cases according to Bethesda category in CS preparation. CS: conventional smear; TBSRTC: the Bethesda System of Reporting Thyroid Cytopathology

Category	TBRSTC category	Cases in number (n = 250)	Cases in Percentage
I	Unsatisfactory	13	5.2
II	Benign	203	81.2
	Colloid cyst/Colloid goiter	179	71.6
	Colloid goiter with hyperplastic nodule	4	1.6
	Lymphocytic thyroiditis	20	8
III	Atypia of undetermined significance/Follicular lesion of undetermined significance	4	1.6
IV	Follicular neoplasm/Suspicious of follicular neoplasm	14	5.6
	Follicular neoplasm	13	5.2
	Hurthle cell neoplasm	1	0.4
V	Suspicious for malignancy	3	1.2
VI	Malignant	13	5.2
	Papillary carcinoma	8	3.2
	Medullary carcinoma	1	0.4
	Anaplastic carcinoma	4	1.6

Both CS and LBC smears were compared for cellular morphology and other features, as shown in Table 3. Overall cellularity of CSs was better than LBC smears. CSs showed score 2 and score 3 cellularity in 190 and 40 cases, respectively, whereas LBC smears had score 2 and score 3 in 163 and 21 cases, respectively. The proportion of specimens obtaining scores 2 or 3 for characteristics, cell architectures, nuclear details, and cytoplasmic details was higher for CSs compared to LBC smears (p < 0.001). Among all cases, informative background colloid was preserved better (in CSs 218 cases) compared to LBC smears (165 cases). The LBC technique was more efficient in producing a clean background in 160 cases compared to 14 cases in CSs.

**Table 3:** Comparison between liquid-based and conventional cytology for different cytological features (n = 250). LBC: liquid-based cytology

Characteristic/Technique	Score 0	Score 1	Score 2	Score 3	P-value
Cellularity					
Conventional	13	7	190	40	<0.001
LBC	39	27	163	21	
Cell architecture					
Conventional	15	34	201	N/A	<0.001
LBC	52	164	34	N/A	
Nuclear details					
Conventional	13	158	62	17	<0.001
LBC	41	160	31	18	
Cytoplasmic details					
Conventional	13	162	68	7	<0.001
LBC	41	170	32	7	
Informative background					
Conventional	32	218	N/A	N/A	<0.001
LBC	85	165	N/A	N/A	
Background blood					
Conventional	14	170	56	10	<0.001
LBC	160	70	20	0	

The measure of agreement between LBC and CS, as shown in Table 4, was excellent with an agreement of 214/250 cases (κ = 0.652, p < 0.001).

**Table 4:** Comparison of paired conventional and liquid-based smears according to Bethesda category. κ = 0.652; p < 0.001. Total agreement = (9 + 173 + 3 + 13 + 3 + 13)/250 = 85.6%. LBC: liquid-based cytology

LBC	Conventional						Total
	Category 1	Category 2	Category 3	Category 4	Category 5	Category 6	
Category 1	9	29	1	0	0	0	39 (15.6%)
Category 2	3	173	0	0	0	0	176 (70.4%)
Category 3	1	0	3	0	0	0	4 (1.6%)
Category 4	0	0	0	13	0	0	13 (5.2%)
Category 5	0	1	0	1	3	0	5 (2.0%)
Category 6	0	0	0	0	0	13	13 (5.2%)
Total	13 (5.2%)	203 (81.2%)	4 (1.6%)	14 (5.6%)	3 (1.2%)	13 (5.2%)	250

According to ease of interpretation, as mentioned in Table 5, LBC was found to be equivalent to CS in the majority of cases (n = 156, 62.4%). CS was easier than LBC in 13.6% (34) of cases, and LBC was easier than CS in only 3.2% (eight) of cases. The non-diagnostic rate was higher in LBC by 15.6% (39) cases compared to 5.2% (13) cases in CS.

**Table 5:** Comparison of paired conventional and liquid-based smears according to ease of interpretation. CS: conventional smear; LBC: liquid-based cytology

Category	Number of cases	Percentage
CS = LBC	156	62.4
CS is easier than LBC	34	13.6
LBC is easier than CS	8	3.2
CS – non-diagnostic	13	5.2
LBC – non-diagnostic	39	15.6
Both – non-diagnostic	09	3.6

Out of 250 cases, histopathology diagnosis was available in 96 cases, as shown in Table 6. Among the 96 cases, 11 (11.4%) were diagnosed as malignant (four follicular carcinomas, two papillary carcinomas, two medullary carcinomas, one anaplastic carcinoma, one follicular variant of papillary carcinoma, and one poorly differentiated thyroid carcinoma). The remaining 85 (88.5%) cases were benign lesions (55 cases of colloid goiter, 19 cases of colloid goiter with hyperplastic nodule, five cases of follicular adenoma, and six cases of thyroiditis). A case of atypia of undetermined significance (category III) on cytology was diagnosed as Riedel’s thyroiditis on histopathology. The three cases of category V were diagnosed as poorly differentiated thyroid carcinoma, anaplastic carcinoma, and medullary carcinoma on histopathology.

**Table 6:** Distribution of cases according to histopathological diagnosis (n = 96).

Diagnosis	Number of cases	Percentage
Malignant	11	11.4
Follicular carcinoma	4	4.2
Follicular variant of papillary carcinoma	1	1.0
Anaplastic carcinoma	1	1.0
Medullary carcinoma	2	2.0
Papillary carcinoma	2	2.0
Poorly differentiated thyroid carcinoma	1	1.0
Benign	85	88.5
Colloid goiter/Multinodular goiter	55	57.2
Colloid goiter with hyperplastic nodule	19	19.8
Follicular adenoma	5	5.2
Thyroiditis	6	6.2

The sensitivity, specificity, PPV, NPV, and diagnostic accuracy of CS and LBC against histopathological examination are presented in Table 7. The sensitivity of CS was 86.4% compared to 68.7% in LBC preparations and specificity was 94.4% and 92.4%, respectively. The diagnostic accuracy of CS was 96.8% compared to 91.7% in LBC. Conventional cytology had PPV and NPV of 68.7% and 97.8%, respectively. LBC had only PPV and NPV of 61.1% and 94.4%, respectively.

**Table 7:** The sensitivity, specificity, PPV, NPV, and diagnostic accuracy of CS and LBC against histopathological examination. CS: conventional smear; LBC: liquid-based cytology; PPV: positive predictive value; NPV: negative predictive value

Technique	Sensitivity	Specificity	PPV	NPV	Accuracy
CSs	86.4%	94.4%	68.7%	97.8%	96.8%
LBC smears	68.7%	92.4%	61.1%	94.4%	91.7%

## Discussion

FNA cytology plays an important role in determining the mode of treatment of thyroid nodules. The Bethesda system for reporting thyroid cytopathology allows standardization and improved diagnostic terminologies between pathologists and clinicians. Controversy exists about the efficacy of LBC smears and CSs for the evaluation of thyroid lesions.

This study comprised 250 cases of thyroid lesions and aimed to evaluate cytomorphological differences between LBC smears and corresponding CSs. The cases were categorized per the Bethesda system. The level of agreement between LBC smears and CSs in this study was (85.6% cases) excellent (N = 250, κ = 0.652). This finding was very similar to Jung et al. and Gupta et al., who reported that the overall agreement of LBC and CS was good (N = 193, κ = 0.687 [7] and N = 60, κ = 0.734 [8], respectively).

**Figure 1:**
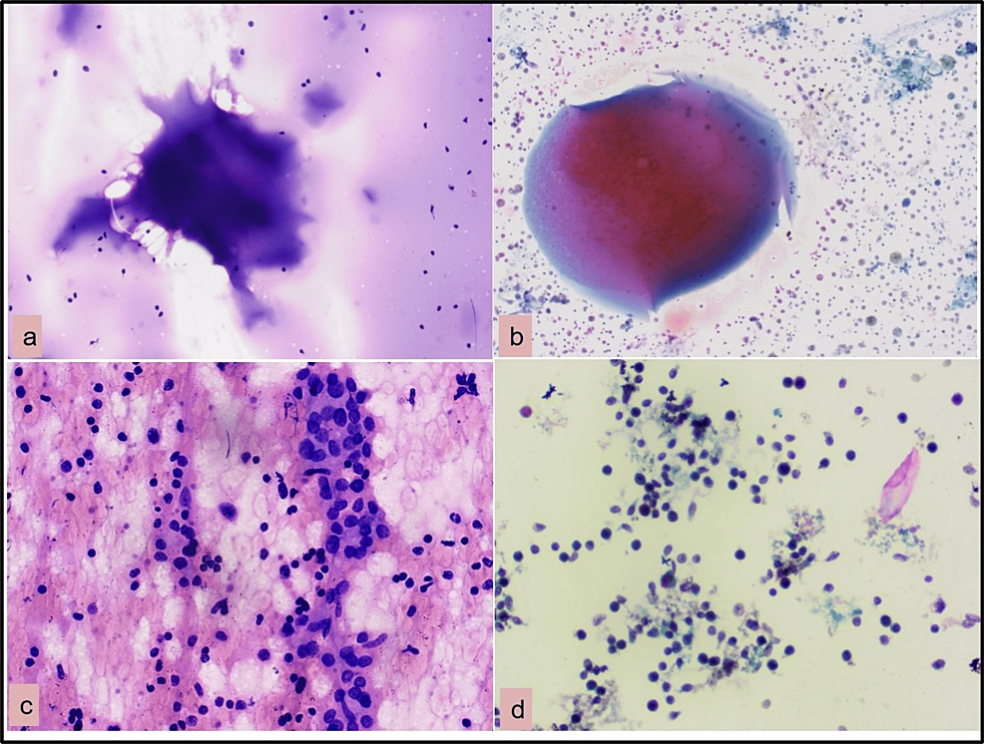
CSs versus LBC smears: colloid goiter (a) showing abundant colloid (H&E 100×) (b) colloid with hemosiderin-laden macrophages (Papanicolaou stain 100×). Lymphocytic thyroiditis: lymphocytes impinging on follicular cells (H&E 200×). (d) Polymorphic lymphoid cells and plasma cells in the background (Papanicolaou stain 200×). CS: conventional smears; LBC: liquid-based cytology; H&E: hematoxylin and eosin

In this study, the maximum cases (81.2%) were benign lesions (category II), followed by follicular neoplasm (category IV) and malignant (category V) (5.2%) each, which represents a low malignancy rate in thyroid lesions. The overall inadequacy rate for CS was low (13 cases, 5.2%) compared to LBC (39 cases, 15.6%). In total, 26 cases of colloid goiter, two cases of lymphocytic thyroiditis, one case of Riedel’s thyroiditis, and one case of hyperplastic nodule were categorized as non-diagnostic by LBC preparation. Colloid goiter was more easily diagnosed with CS (179 cases) than LBC (153 cases) due to the presence of an informative background. In LBC, colloids appeared as small droplets or as napkin folds and cyst macrophages were more pronounced in LBC due to diminished blood and a clean background (Figure 1, Panels a and b). The inadequacy rate of LBC has been variably reported in the literature; 11.2% reported by Sharma et al. [9] 18% by Mahajan et al. [10], and 25% by Geers et al. and Cochand et al. [11,12]. INagarajan et al. [13] observed that LBC was associated with a significantly higher proportion of inadequate cases. Because we had poured FNA material of only one pass into the LBC vial and one slide made by BD SurePath automated machine, the high inadequacy rate in LBC may be due to the dilution of cells in the suspension medium; second, colloid was also reduced/lost due to processing. This was supported by Sharma et al. [9], who found that in LBC preparation a repeat slide preparation from the residual material made some of the cases adequate, and a proper diagnosis could be made.

Further, non-diagnostic cases were maximum in the benign group (category II) where informative background was lacking and follicular cells were scattered singly rather than follicles making diagnosis difficult. Hence, in the present study, ease of interpretation was greater with CSs compared to LBC smears, especially in colloid goiters/ colloid cysts. In the CS preparation, cytopathologists have the opportunity to ensure the adequacy of diagnostic material by directly seeing brownish fluid colloid and some granularity during the spreading of material on the slide. In this study, 20 cases of lymphocytic thyroiditis CS and LBC were equivalent and LBC had the advantage of dispersed inflammatory cells in a clean background, while CS had a relatively greater number of inflammatory cells, giant cells, and better visualization of lymphocytes impinging on follicles (Figure 1, Panels c and d).

**Figure 2:**
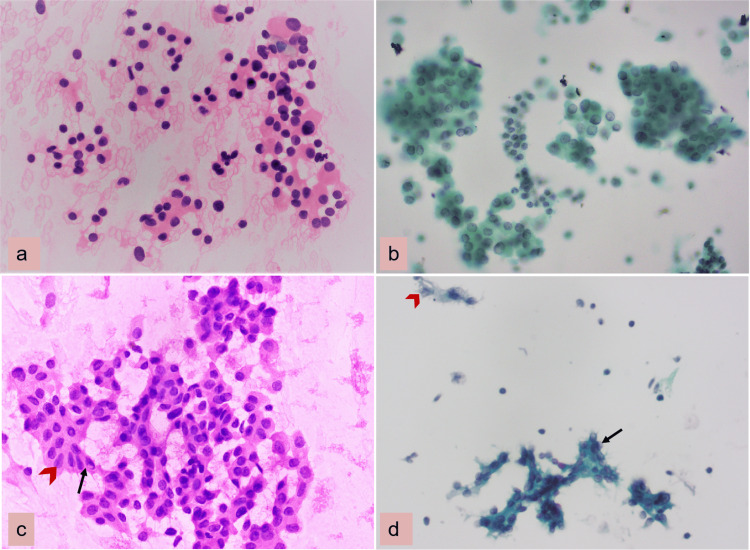
CSs versus LBC smears: (a) Follicular neoplasm showing overlapping micro follicles with mild anisonucleosis (H&E 400×). (b) Papanicolaou stain 400×. (c) Papillary thyroid carcinoma showing intranuclear inclusions (arrow) and elongation of nuclei (arrowhead) (H&E 400×). (d) Pale nuclear chromatin (arrow) irregular nuclear membrane with groove (arrowhead) (Papanicolaou stain 400×). CS: conventional smears; LBC: liquid-based cytology; H&E: hematoxylin and eosin

Category IV, follicular neoplasms, showed fewer micro follicles and more naked nuclei compared to CS preparation (Figure 2, Panels a and b). Similar observations were also noticed by Mahajan et al. [10], Sharma et al. [14], and Kumari et al. [15]. Moreover, the LBC preparation had a higher frequency of suspicion for malignancy cases (five) than CS (three). This may be due to the high N:C ratio and overlapping cluster looking more hyperchromatic and fragile cytoplasm in LBC cases. This observation was similar to Kumari et al. [15], who also reported overdiagnosis of malignancy on LBC as most of the cases were categorized as suspicious for malignancy (19 cases in LBC vs. 4 cases in CS).

In category VI, CS and LBC showed equal ease of interpretation and had a score of 3+ cellularity in most cases. In malignant cases, thyroid nodule was more cellular and FNA yielded good cellularity. Cytomorphological features in papillary carcinoma such as oval, overlapping nuclei, powdery chromatin, and nuclear grooves were also well appreciated in LBC smears (Figure, Panels c and d).

**Figure 3:**
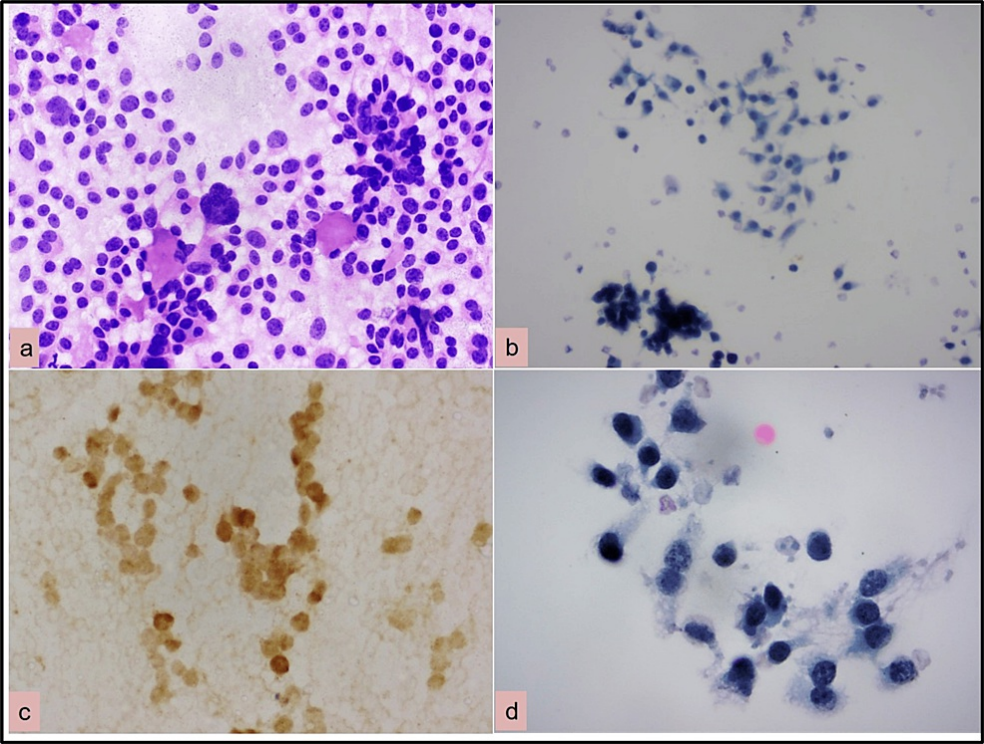
CSs versus LBC smears: Medullary thyroid carcinoma (a) showing round, plasmacytoid, spindle cells in nests, or follicles with amyloid (H&E 200×). (b) Round to spindle cells (Papanicolaou stain 200×). (c) Calcitonin diffusely positive in tumor cells (400×). (d) Tumor cells with finely stippled chromatin (Papanicolaou stain 1,000×). CS: conventional smears; LBC: liquid-based cytology; H&E: hematoxylin and eosin

Intranuclear inclusions were noticed less frequently. Similar features were observed by many authors [8-10,16,17]. In the case of medullary carcinoma, amyloid was better visualized in CS while salt paper chromatin character was well recognized in LBC (Figures 3a, 3b, 3d). The second slide was made from residual material and calcitonin was determined which was positive and confirmed the diagnosis of medullary carcinoma (Figure 3c).

In cases of anaplastic carcinoma, LBC was good as CS prominent nucleoli and coarse chromatin were seen.

However, there were fewer malignant cases in our study, and the role of immunocytochemistry and molecular studies in follicular lesions needs to be explored further.

According to ease of interpretation, LBC was as good as CS in 62.4% of cases, and only in 3.2% of cases it was better than CS. Mahajan et al. [10] reported similar findings where LBC was only as good as CS in 58% of cases and was better than CS in 5% of cases.

In this study, the sensitivity of CS was 84.6% compared to 68.7% in LBC preparations. Diagnostic accuracy of CS and LBC were 96.8% and 91.7%, respectively. Kumari et al. [15] studied 100 cases and found that the diagnostic accuracy of CS and LBC was 100% and 40.7%, respectively, for surgically resected cases. Cavaliere et al. [16] also observed higher sensitivity in CS compared to LBC smears (93.6% vs. 65.9%). In the study of Chang et al. [18], the sensitivity of CS and LBC was 78.9% and 76.3%, respectively.

In contrast to these findings, Jung et al. [7] found a higher sensitivity of FNA in diagnosing thyroid neoplasm at 93.9% vs. 90.9% for LBC and CS, respectively. LBC slides showed increased cellularity and more clustered tissue fragments.

Sayer et al. [19] concluded that there was no statistical difference between the results of both methods while classifying thyroid nodules according to Bethesda category II to VI, but the non-diagnostic biopsy rate was higher in the specimens prepared by the CS method (p < 0.001). In their study, ultrasonography-guided FNA was performed and slides were prepared by either the CS or LBC method without an accompanying cytopathologist during the procedure. In our opinion, on the site of FNA, a cytopathologist plays an important role in ensuring the quality of fluid and the type and adequacy of material along with the first perception of the lesion.

In our study, the overall diagnostic efficacy was better for CS; hence, we favor CS for benign lesions. LBC and CS can be done together to enhance diagnostic efficacy in malignant and suspicious thyroid nodules. Ardito et al. [20] also reported that the use of CS and LBC together allows the number of unnecessary thyroidectomies to be reduced. Cochand et al. [12] also reported that LBC was not useful in goiter and infectious lesions. Similarly, for malignant thyroid lesions, Saleh et al. [21] also found better results in anaplastic and medullary carcinoma in LBC preparation.

## Conclusions

CSs were easier and inexpensive to prepare, and LBC smears had lower sensitivity and higher non-diagnostic rates than CSs. Hence, CSs would not be replaced by LBC smears in developing countries where cost is the prime factor for the majority of the patients. For benign lesions of the thyroid, we recommend CSs. LBC is a good adjunct to CS in diagnosing malignant/suspicious thyroid lesions as it provides excellent nuclear and cytoplasmic details in a clear background and reuses residual material for ancillary testing to prevent re-aspiration.
